# Progress in diagnostic methods and vaccines for lumpy skin disease virus: a path towards understanding the disease

**DOI:** 10.1007/s11259-025-10667-2

**Published:** 2025-03-08

**Authors:** Tarek Korany Farag, Hala A. A. Abou-Zeina, Sobhy Abdel-Shafy, Ahmad M. Allam, Alaa A. Ghazy

**Affiliations:** Department of Parasitology and Animal Diseases, Veterinary Research Institute, National Research Centre, Giza, Egypt

**Keywords:** Lumpy skin disease virus, Lumpy skin disease, Arbovirus infections, Vaccination, Livestock

## Abstract

Lumpy skin disease (LSD) is caused by Lumpy Skin disease virus (LSDV) belonging to the genus *Capripoxvirus* (CaPV). The disease is widespread in Africa, the Middle East and Asia and has been present in Egypt since 1988. LSD is mainly transmitted by blood-sucking insects. LSD is clinically distinguished by a high fever, skin nodules, and swollen Lymph nodes. Detecting sub-clinical disease can be challenging however, prompt laboratory investigations are vital. Skin lesions are the main source of infection, although the virus is shed through many excretions and discharges including semen. Disease confirmation in clinical laboratories includes detection of viral nucleic acid, antigen and antibody levels. Simple, adaptable, and quick assays for detecting LSDV are required for control measures. Vaccination, together with controlled quarantine and vector control measures, may be beneficial for preventing disease spread. Presently, a range of live attenuated vaccines, have been used in the field with different levels of protection and side effects. With high levels of vaccination coverage, attenuated Neethling vaccines have successfully eradicated of LSDV in Europe. Inactivated LSDV vaccines have also been demonstrated effective in experimental infections. Furthermore, due to its large genome, LSDV is being exploited as a vaccine delivery element, generating an innovative composite with additional viral genes by DNA recombination. Vaccines developed on this basis have the potential to prevent a wide range of diseases and have been demonstrated to be effective in experimental settings. In this review, we emphasizethe advances in diagnostic methods and vaccines developed last decade, thereby providing a basis for future research into various aspects of LSDV and providing information for possibility of disease elimination.

## Background

Lumpy skin disease is a transboundary emerging viral disease. Cattle are considered the main host of LSDV. Recently, LSDV was detected in camels, with a low incidence in buffalos (Modise et al. 2021). LSDV is an enveloped double-stranded DNA virus, along with sheep poxvirus (SPPV) and goat poxvirus (GTPV), which constitute the genus CaPV of the *Poxviridae* family (Diallo and Viljoen 2007). LSDV is of unknown origin. Zambia was the country where the virus was firstly reported in the world in 1929. The disease was firstly diagnosed as an insect bite hypersensitivity reaction in cattle (Shalaby et al. 2016). LSDV has spread throughout Sub-saharan Africa, then the Middle East, southern Europe, North Africa and Asia. The disease negatively impact the growth of cattle and buffalo production (Ayelet et al. 2014).

In Egypt, LSDV was first detected in the Governorate of Suez Canal in the summer of 1988 and subsequently spread through Egypt. It infected 50,000 cattle and caused mortality in 1,449 cattle in 1998 (Salib and Osman 2011; Ali et al. 2021). During the 1988 LSD epidemic in Ismailia, the disease appeared only in cattle, while sheep, goats, and water buffaloes seemed apparently healthy (House et al. 1990).

The disease has low mortality and an extraordinary morbidity rate and affects livestock of all ages and breeds (Namazi and Khodakaram Tafti 2021). Epidemics of LSD are infrequent since the virus relies on the movements and immune status of animals, and wind and rainfall play a crucial role in the vector ecosystem (Machado et al. 2019). LSDV can spread in several ways, for instance, presence of susceptible animals in the same vicinity of vectors, eye secretions, semen, milk, and saliva (Hailu et al. 2014; EFSA 2018). As a result of the rapid spread of LSDV and resulting in severe fatalities, the World Organisation for Animal Health (WOAH), formerly the *Office International des Epizooties* (OIE)listed LSDV as a notifiable cattle disease (Bowden et al. 2008).

The diagnosis of LSD initially depends on clinical signs. The clinical disease is variable with acute, and suc-clinical infection chronic (Hailu et al. 2014). The most common clinical manifestations are fever, skin nodules, emaciation, swollen lymph nodes, skin edema and death (Molla et al. 2017; Farag et al. 2020). There is no advised medication for LSD. Nevertheless, symptomatic approaches for diseased animals may be used to counteract secondary bacterial infections (Datten et al. 2023). Following recovery from LSD, animals can suffer from longstanding udder and lung inflammation and badly defective hides (Selim et al. 2021).

The accessibility and superiority of diagnostic devices are often ideal factors for the effectiveness of disease control, extinction, or avoidance. Unmistakable diagnosis can be accomplished via virus isolation, electron microscopy (EM), antigen detection by immunofluorescence (IF), serum neutralization test (SNT), agar gel immune diffusion (AGPT), antigen capture Enzyme-Linked Immunosorbent Assay (ELISA) and Dot ELISA (Tuppurainen et al. 2021). Detection of low antibody titers after infection using serological techniques, such as the virus neutralization test (VNT), is not very sensitive and can be time-consuming (WOAH 2023).

Only one ELISA kit has recently been commercialized, and while the first findings are promising, its actual potential must yet be verified in the field (Haegeman et al. 2021). In addition, conventional and real-time PCRs for the detection of *capripox virus* (CaPV), sheep poxvirus (SSPV), goat poxvirus (GTPV) and LSDV have been described with associated high sensitivity and specificity, (Shalaby et al. 2016; Armson et al. 2017). Additional molecular diagnostics including conventional and real time PCR can distinguish between the three species LSDV, SSPV and GTPV (Molini et al. 2018). LSDV-recombinase polymerase amplification (RPA), loop isothermal amplification (LAMP) and Triple*E* (*E*asy-*E*xpress-*E*xtraction) assays as a fast and affordable method for nucleic acid and isolation method centrifugation-free and electricity-free can be helpful because of their rapid, excellent accuracy and sensitivity, which can be achieved in the field or at quarantine location diagnostic laboratories (Mwanandota et al. 2018; Chala 2022; Jiang et al. 2022; Korthase et al. 2022).

Vaccination, together with controlled quarantine procedures and vector control, may be beneficial for preventing disease transmission (EFSA 2017; WOAH 2023). LSD control and prevention rely heavily on vaccination (Klement et al. 2020). Sheeppox virus (SPPV) and goatpox virus (GTPV) share antigenic homology and cross protection with LSDV, vaccinations using live attenuated SPPV and GTPV vaccines have been used in the field with varying success to control LSD. There are some issued with using live attenuated vaccines especially if the virus is not fully attenuated or if there is poor quality control in production of the vaccine with contaminating viruses (Liu et al. 2021; Tuppurainen et al. 2021). Despite the Egyptian government's yearly mass inoculation with the sheeppox (SPP) vaccine (Veterinary Serum and Vaccine Research Institute, VSVRI, Egypt), LSDV still spreads practically every summer. For example, a major LSD outbreak swept Egypt in the summer of 2006, affected 16 provinces, and then resurfaced in 2011 and 2014 (El-Tholoth and El-Kenawy 2015).

Therefore, this review aims to highlight current research advancements in diagnostic methods and vaccine types, laying the groundwork for future studies on various aspects of LSDV and providing information for disease eradication.

## Etiology

The viruses that cause LSD belong to the order *Chitovirales*, family *Poxviridae* and genus *Capripoxvirus* (Farah Gumbe 2018). The SPPV, GTPV and LSDV all belong to the same genus but are phylogenetically distinct from each other. There is only one known serotype of LSDV (Şevik and Doğan 2016). LSDV is a double-stranded DNA virus with a 151-kbp genome (Bhanuprakash et al. 2006).

LSDV is stable and can survive in organic materials for a long period of time; LSDV can be found in skin nodules even after 30 days. The virus is susceptible to even mild chemical modification with either an alkaline or acidic pH, which could reduce the viral load. Ether, chloroform, formalin, phenol and other substances may also reduce the viral load. The virus titer remains unchanged after exposure to a pH range of ≥ 6.5 to ≤ 8.5 for four to seven days at 37 °C (WOAH 2023).

## Occurrence

CaPV infections have definite geographic dispersals (Davies 1991; Mulatu and Feyisa 2018). SPPV and GTPV are prevalent in most African countries, the Middle East, Central Asia and the Indian subcontinent. In contrast, LSDV affects most African countries (Bhanuprakash et al. 2006; Lefèvre et al. 2010). Its incidence in Egypt was confirmed between 1988 and 1989, and it was re-emerged again in 2014 in buffaloes (Elhaig et al. 2017). LSD have also been reported on the European and Asian continents (Al-Salihi and Hassan 2015; Mulatu and Feyisa 2018; Gupta et al. 2020; Sethi et al. 2022). In 2015 and 2016, the disease spread to Southeast European countries (WOAH 2023). LSDV outbreaks are more frequent throughout the rainy season, especially in low-lying locations or near water; however, LSDV can also arise during the drought season (Gelaye and Lamien 2019). LSD outbreaks tend to be intermittent since viruses rely on the movements and immune status of animals, and wind and rainfall play crucial roles in vector ecosystem (Machado et al. 2019).

## Transmission

Blood-feeding insects such as mosquitos and flies, as well as ticks, which play a role as mechanical vectors, distribute the disease at distances greater than 50 km from the adjoining recognized disease focus (WOAH 2023). The mechanical transmission of the virus was shown to be efficient by *Aedes aegypti*, where susceptible cattle can acquire the disease through biting of carrying mosquitoes that are able to transmit the virus for 2–6 days (Chihota et al. 2001). Diverse routes, such as blood, discharge, semen and saliva, can be used as transmissible aids. The disease can also be transferred to nursing calves when they are fed infected milk or from sores on their mothers’ teats (Tuppurainen et al. 2017). The transfer through artificial insemination was recorded from infected bull semen to the heifer as well as inherently to the fetus (Carn and Kitching 1995; Irons et al. 2005). Experimental laboratory work and field studies have proven the probable spread of LSDV via direct contact, which has a low incidence (Namazi and Khodakaram Tafti 2021). However, experimental infection can cause disease in animals inoculated with nodules or even blood (Carn and Kitching 1995; Chihota et al. 2001). In these experimental cattle, LSDV was detected 11, 22 and 33 days after the onset of fever in saliva, semen, and nodules, respectively. Similar to other poxvirus families, which are known to be highly resilient, LSDV can persist in infected tissue for more than 120 days (Swami and Verma 2022). Control measures against LSD include disinfection of contaminated facilities, immunization, vector control, identification of infected animals, proper carcass disposal, quarantines, and movement control. Shumilova et al. (2024) studied the transmission of the LSDV from subclinical infected animals through inoculation of healthy non-vaccinated Russian black pied bulls with the recombinant vaccine-like strain (RVLS) Udmurtiya/2019 to induce subclinical LSDV infection. After the disease appeared, the subclinically ill animals were reallocated to new facility followed by the introduction of another five healthy animals. Two introduced animals contracted the virus, showing typical symptoms and seroconversion in one animal, while three other introduced animals remained healthy and PCR-negative until the end of the study. On the other hand, transmission of the LSDV through *Stomoxys calcitrans* flies was studied and concluded its role in transmission of LSDV from subclinical infected animals (Haegeman et al. 2023).

## Diagnosis of LSD

**Fig. 1:**
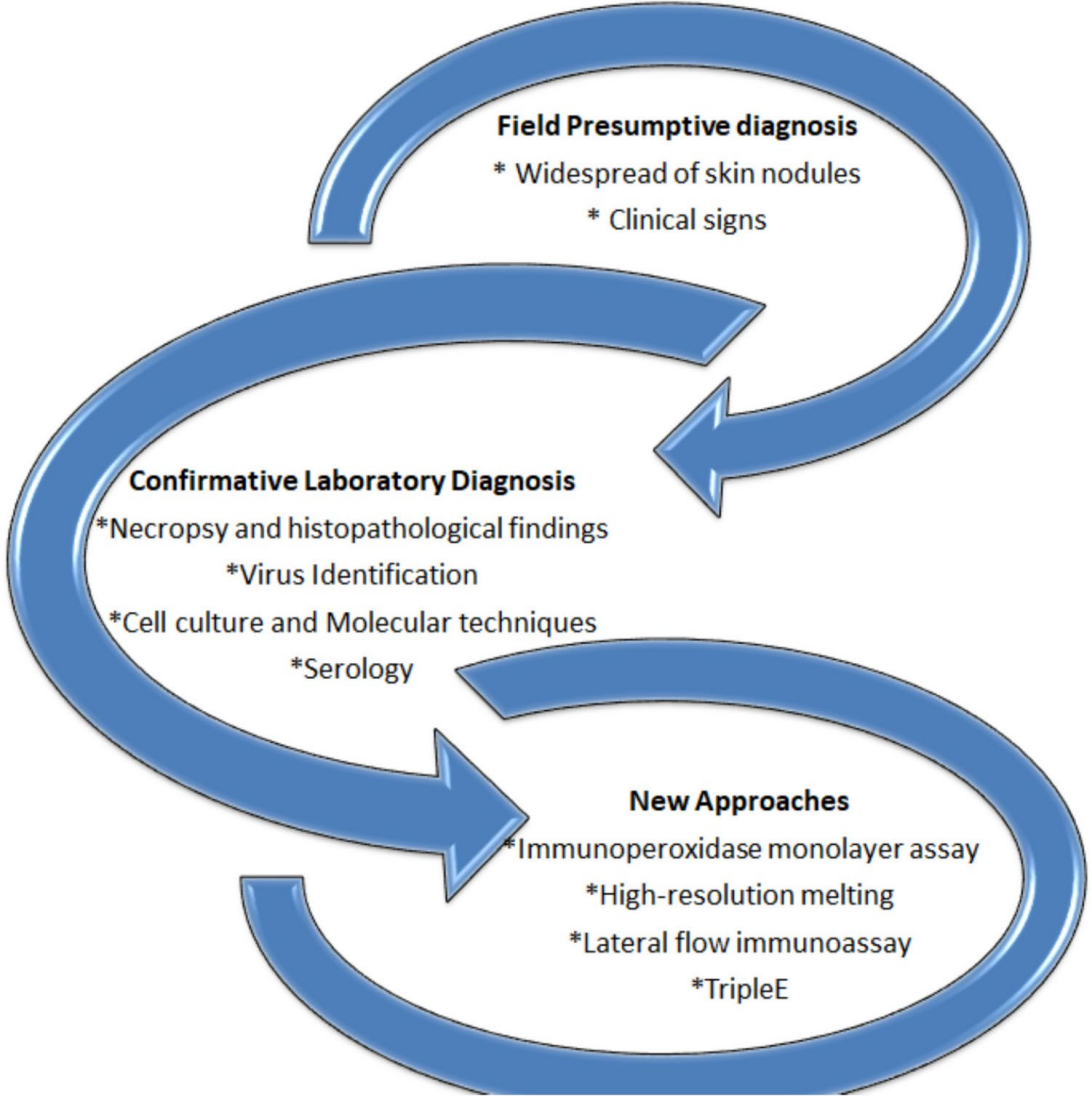
The diagnostic methods for LSDV

The diagnosis of LSD relies on two primary foundations: first, the observation of identical clinical signs, and second, laboratory confirmation of the virus's presence either through basic laboratory assays and/or new modern approaches (Fig. 1 and Table 1).

**Table 1:** Diagnostic methods for LSDV (Liang et al. 2022)

Method	Targets	Accuracy	References
Molecular Methods			
Polymerase chain reaction (PCR)`	P32 gene	98%	(Heine et al. 1999)
	LSDV nucleic acid	Skin samples 100% Blood samples 77.8%	(Awad et al. 2009)
	LSDV nucleic acid	Skin samples 34.78% Blood samples 28.26%	(Zeedan et al. 2019)
Real-time PCR (qPCR)	ORF074 gene	Unknown	(Babiuk et al. 2008)
	LSDV nucleic acid	Skin samples 39.13% Blood samples 36.95%	(Zeedan et al. 2019)
Recombinase polymerase amplification (RPA) assay	LSDV nucleic acid	100%	(Shalaby et al. 2016)
Real-time high-resolution fusing PCR	LSDV-ORF010	Unknown	(Pestova et al. 2018)
Loop-mediated isothermal amplification (LAMP)	VP39 gene	68.42%	(Mwanandota et al. 2018)
Nanopore sequencing	RPO30, P32 and GPCR	Unknown	(Eltom et al. 2021)
Triple*E*	LSDV nucleic acid	Unknown	(Korthase et al. 2022)
Pathological approach			
Pathological section examination	Intracytoplasmic inclusion bodies	Unknown	(Ali et al. 1990)
Histopathological examination	Skin pathology section	Unknown	(Sanz-Bernardo et al. 2020; Ali et al. 2021)
Immuno-assays			
Fluorescent antibody technique (FAT)	LSDV protein	Skin samples 26.08% Blood samples 32.60%	(Zeedan et al. 2019)
Indirect Enzyme-linked immunosorbent assay (iELISA)	LSDV antibodies	17.93%	(Zeedan et al. 2019)
Indirect FAT (iFAT)	LSDV antibodies	14.48%	(Zeedan et al. 2019)
Immunoperoxidase monolayer assay (IPMA)	LSDV antibodies	100%	(Haegeman et al. 2020)
Enzyme-linked immunosorbent assay (ELISA)	LSDV-specific antibodies in milk	Unknown	(Milovanović et al. 2020)
High-resolution melting (HRM)	PCR amplicons of samples	Unknown	(Modise et al. 2021)
Immunohistochemical (IHC)	LSDV antigen	Unknown	(Ali et al. 2021)
RPA-Cas12a-fluorescence assay	ORF068 gene	96.3%	(Jiang et al. 2022)
Lateral flow immunoassay (LFIA)	P32 gene	Unknown	(Cavalera et al. 2022)
Standard techniques			
Trials for VI and identification on ECEs	LSDV	Unknown	(Ali et al. 2021)

### Field presumption diagnosis

The field presumption diagnosis of LSD can be based on the following:

A. Morbidity and mortality

The morbidity rate diverges amid 5 to 45% (occasionally reaching 100%). However, the mortality rate is typically less than 10% (may reach 40%). The severity of clinical disease can be affected by factors such as age, breed, and the body condition of the animal, in addition to the duration of the housing and fattening period (Namazi and Khodakaram Tafti 2021).

B. Clinical signs

The incubation period of the disease ranges from 1 to 4 weeks, fever develops, and finally, the infected animal becomes despondent, which continues for approximately 4 to 14 days (Das et al. 2021). The clinical picture of LSD may differ from that of acute, subacute, or inapparent LSD. Ideal LSD is distinguished by high body temperature (> 40.5°C) and nodules (10–50 mm diameter), which are dreadful and hyperemic, hitting other organs, such as the ear, muzzle, cranium, eyelids, teats, limbs, perineum, and genitalia (Hailu et al. 2014).

The nodules are wide, elevated protrusions and somewhat from adjoining skin isolated by hemorrhagic rings. The nodules involved the dermis, epidermis, adjacent subcutis and musculature. Consequently, the nodules develop into papules, vesicles, and pustules with exudation, and then a scab develops gradually. After 2–3 weeks, the cutaneous lesions become firmer and necrotic, and sloughing of the lesions may produce “sitfast” holes, the distinguishing lesion, which may subsequently be invaded by the screwworm fly as well as bacterial contamination that can lead to additional septicaemia (Shrirame et al. 2023).

**Fig. 2:**
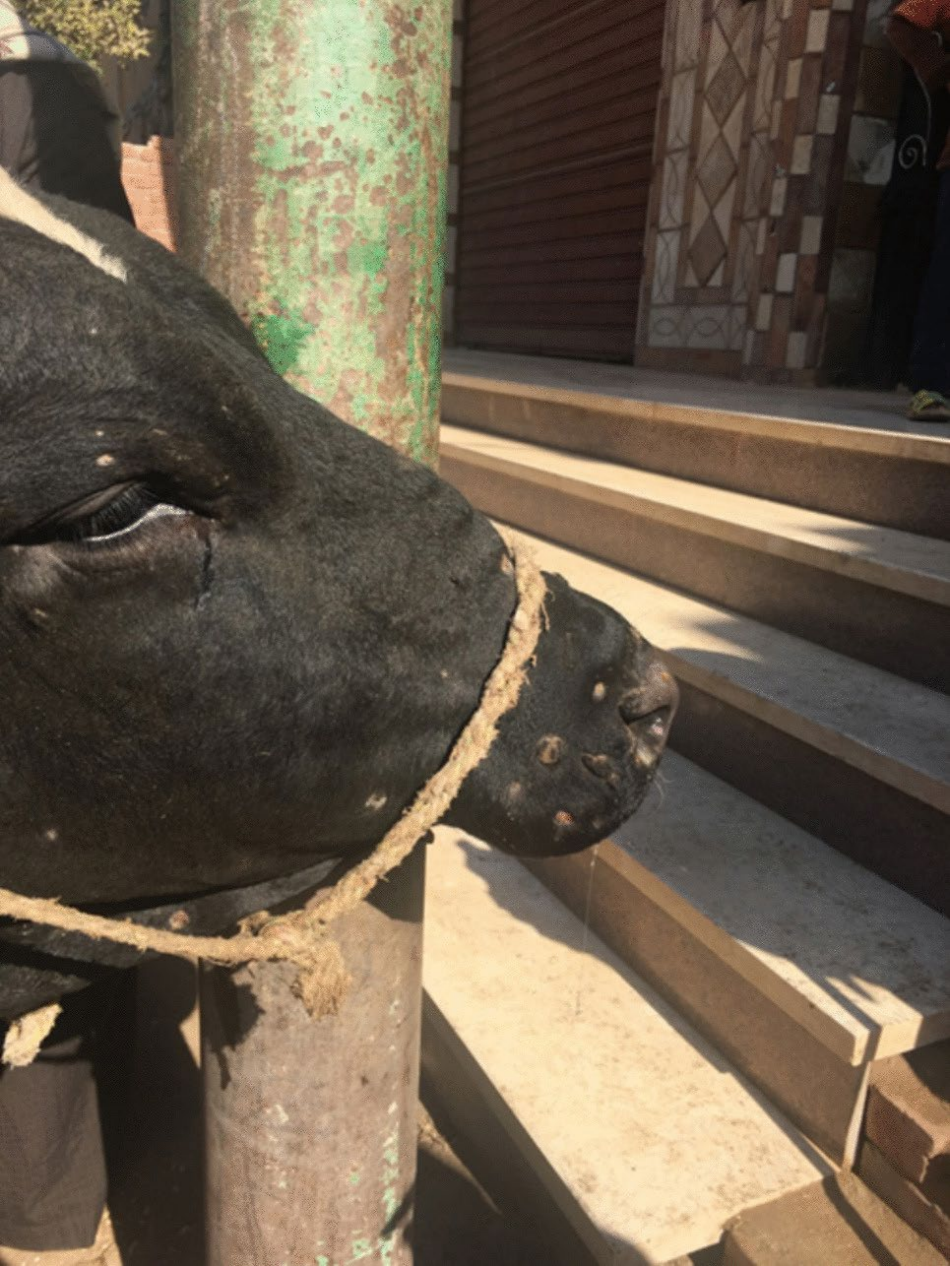
Cattle head showing lumpy skin disease infection scars in the late stage (Allam et al. 2020)

**Fig. 3:**
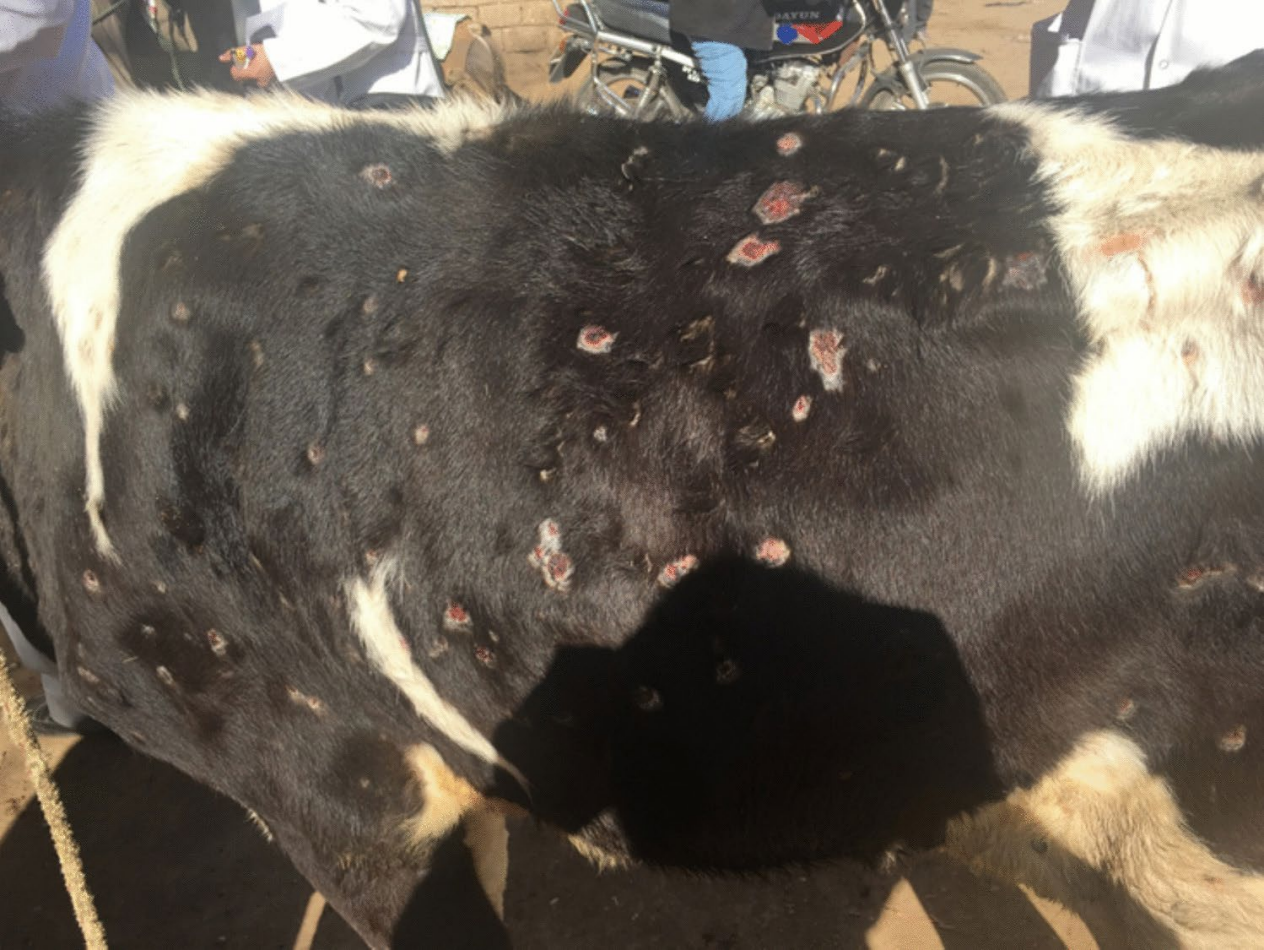
Calf skin showing lumpy skin disease infection scars in the late stage (Allam et al. 2020)

**Fig. 4:**
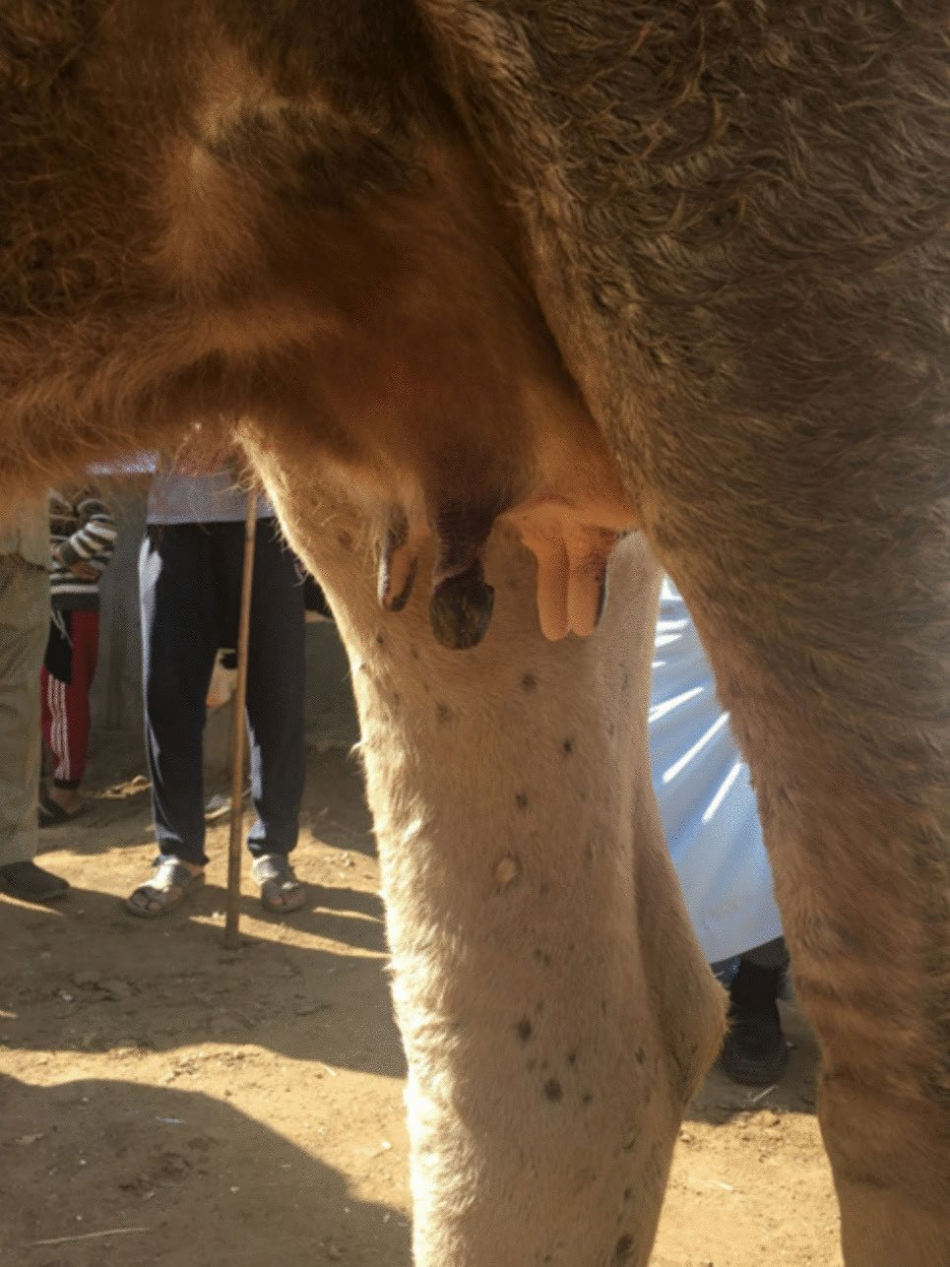
Cow teat showing complete dryness of one teat due to lumpy skin disease infection (Allam et al. 2020)

Further clinical signs include lachrymation and nasal discharge; the presence of enlarged lymph nodes, such as prefemoral and subscapular ones; and decreased milk return (Farah Gumbe 2018). Inflammatory and edematous enlargements of the limbs, brisket, genitalia, and face may develop. The complications of LSD include pneumonia caused by the inhalation of necrotic debris, abortion during the acute phase of infection, infertility in both males and females, emaciation, and lameness (Shrirame et al. 2023) (Figs. 2, 3 and 4).

### Confirmative laboratory diagnosis of LSD

#### Pathology

##### Necropsy findings and histopathological examination

In autopsies, lung edema and congestion, nodules throughout the lungs and gastrointestinal tract and enlarged superficial lymph nodes are often observed (Zeynalova et al. 2016). Small nodules, such as pox bumps, can be observed in the mucous membrane throughout the body (Rouby et al. 2021). The consequences of severe disease may include keratitis, dysentery, pneumonia, mastitis and myiasis (Al-Salihi and Hassan 2015; Tuppurainen et al. 2017).

Histopathological results of LSD are distinctive and basic for diagnosis. Various studies have described in detail the ideal biopsy picture of LSD skin nodules (Rouby et al. 2021; Akther et al. 2023; Shumilova et al. 2023). The tissue showed a range of pathological changes, such as acanthosis and vacuolar degeneration, in the epidermal layer. Some skin nodules showed dilatation of the lymph vessels (LVs), vasculitis and leucocytic infiltration. The dermal layer showed substantial leucocyte infiltration of eosinophils. Chromatin condensation and severe hemorrhages with leucocytic aggregation were observed. The necrosis and dissociation of muscle fibers, fatty infiltrates and leucocytic infiltration were obvious. Red viral particles were detected in the macrophages of the connective tissue of the dermal layer using immunohistochemistry.

##### Hematological and serum biochemical changes

Recent research has involved hematological and biochemical assessments of animals naturally and empirically infected with LSDV (Abutarbush 2015; Neamat-Allah 2015; Şevik et al. 2016). Research has shown that there is a substantial decrease in most blood count elements with a marked increase in the mean corpuscular volume in experimentally infected animals, which is translated to macrocytic hypochromic anemia (Neamat-Allah 2015). However, leucogram results demonstrated leucopenia and lymphopenia, possibly due to viral infection and granulocytic leukocytosis, which could be due to secondary acute bacterial infections. LSD is also accompanied by thrombocytopenia, hyperfibrinogenemia, decreased creatinine concentration, hyperchloremia and hyperkalemia in naturally infected cattle (Abutarbush 2015). Studies have shown a noteworthy decrease in total protein and serum ALB; however, there was a substantial increase in globulin, especially gamma globulins, in LSD-infected cows (Abutarbush 2015; Neamat-Allah 2015). In addition, serum biochemical analyses of LSD-infected cattle revealed that aspartate aminotransferase and alkaline phosphatase increased in addition to globulin protein and creatinine (Şevik et al. 2016). The studies suggested that the changes in the serum biochemical parameters might be due to liver and kidney failure, severe inflammatory processes, and disease complications, such as anorexia and reduced muscle mass during LSDV infection.

#### Agent identification

##### Isolation of the virus

LSD virus isolation is considered a feasible way to confirm the disease in a new area. The first week of clinical symptom incidence is considered the targeted time at which samples are collected for virus isolation before the surge of neutralizing antibodies (Davies and Otema 1981; Davies 1991). Skin biopsies of skin lesions contain the high levels of virus for virus isolation and transmission via electron microscopy (EM). In addition, buffy coats separated from heparinized or ethylenediaminetetraacetic acid (EDTA) blood samples can be used for isolating LSD virus during the viremic phase of the disease. Virus can also be isolated from enlarged lymph nodes. Cell culture of bovines, ovines or caprines would be highly beneficial for the growth of LSD virus. For instance, bovine dermis cells OA3.Ts, ESH-L or lamb testis (LT) cells (primary or secondary culture) are deemed to be the most susceptible cells. The virus has also been adapted to grow on the chorioallantoic membranes (CAMs) of embryonated chicken eggs (ECEs) and African green monkey kidney cells, which is not recommended for primary isolation (WOAH 2023).

##### Transmission electron microscopy (TEM)

TEM for the diagnosis of LSD is one of the fastest methods for confirming the presence of the virus within a few hours of sample delivery where it can be utilized to classify the classic poxvirus strain (WOAH 2023). By using phosphotungstic acid (negative stain) of biopsy specimens collected from skin nodules or mucous membranes and inoculated on MDBK cell culture media, TEM revealed the typical outline of the virus particle as an ovoid shape, a rounded ending bilayer, and a ball of wool filaments (Aboelkhair et al. 2019; Tran et al. 2023). Mature capripox virions have regular dimensions of 320 × 260 nm and oval bodies with larger lateral edges than orthopox virions (Aboelkhair et al. 2019).

##### Molecular detection methods

Molecular techniques, are crucial for monitoring the spread of viruses and managing disease outbreaks.

A. Polymerase chain reaction (PCR)

Conventional PCR and real-time PCR are considered diagnostic tools for the detection of LSDV outbreaks (Datten et al. 2023). PCR is a very plausible test for diagnosis because of its speed, sensitivity, and specificity (El-Khabaz et al. 2020; Zewdie 2021; Sethi et al. 2022). PCR is a simple, rapid and sensitive system used to detect capripox virus DNA in vast and diverse samples. However,, capripoxvirus assays cannot determine if the virus is LSDV, SSPV or GTPV. Specific primers were designed from the genes of the attachment and fusion proteins of CaPV to detect its genome (Ireland and Binepal 1998). Quantitative real-time PCR has been considered the best tool for diagnosis because it is faster and has better sensitivity (Balinsky et al. 2008; Bowden et al. 2008). The LSD virus genome encompasses 156 recognized genes (Tulman et al. 2001). Advanced molecular procedures such as sequencing and phylogenetic analysis can distinguish strains of the virus (Le Goff et al. 2009). PCR results for samples taken from skin nodules are more positive than those from blood or septic viscera where the virus load is high in the nodules (WOAH 2023). In addition, molecular approaches can aid in determining the behavior of viruses during outbreaks in terms of the production of cytokines, including proinflammatory cytokines, interferons (IFNs) and anti-inflammatory cytokines, and interleukines (ILs) (Tran et al. 2023).

B. Sequencing and Phylogenetic Analysis

Numerous conventional and real-time PCRs have been used to detect CaPV (Venkatesan et al. 2014; Zro et al. 2014), but none are able to differentiate between various species. Due to the prominent similarity of up to 96% between the members of this genus, one PCR assay failed to discriminate between these viruses; however, many GTPVs were classified as SPPVs (Lamien et al. 2011). Sequencing and phylogenetic analysis can be utilized to differentiate between LSDV, SPPV and GTPV. Three genes (RPO30, GPCR, and P32) were analyzed by single-nucleotide polymorphism and nanopore sequencing to determine the differences among the three viruses. The simplicity of repetition in this database yields broad use. These tools are suitable for determining the clinical location, and spreading time (Eltom et al. 2021). (Allam et al. 2020) reported that the genes detected in samples collected from Sohag and Beni-Suef were identified by PCR, and the sequencing findings were 100% identical to those of LSDV. However, the LSDV nucleotide sequence of the Beni-Suef isolate was 100% identical to that of the LSD fusion gene isolated from Giza, Dakahlia, Menufia, Behera, and Beni-Suef throughout the previous decade. The sequences of LSDV isolated from Namibia were similar to those previously detected in African countries such as Egypt, Niger, Burkina Faso and South Africa as well as Eurobian countries such as Greece and Serbia (Molini et al. 2018). This approach aims to increase the detection rate and sensitivity of the poly(A) polymerase small subunit (VP39) gene. It is viable to spot exceptionally small amounts of nucleic acid present via experimental methods. Three positive samples (one from Al-Behera and two from Kafr El-Sheikh) were detected by PCR, and their sequences were found to be strongly linked to the Kazakhstani Kubash/KAZ/16 strain that was previously registered with accession number MN642592. Phylogenetic analysis revealed that the GPCR gene associated with the LSDV strain that was detected in Egypt in the past two years belongs to a universal cluster of LSDV with a similarity of more than 98% (El-Ansary et al. 2022). The present global LSDV state involves the incident in the field of wild-type lineages, vaccine lineages, and at least one vaccine-like recombinant strain (R4) that is consistently spreading in Asia. Although sequencing can help in revealing these new lineages, it is highly recommended to do careful selection and application of phylodynamic approaches, as well as the importance of whole genome sampling in endemic and outbreak areas to improve consideration of the epidemiology, and transmission dynamics of LSDV (Biswas et al. 2020; Van Borm et al. 2023).

C. Loop Isothermal Amplification Assay (LAMP)

Some farms lack expensive and high-precision instruments, such as PCR machines. The LAMP assay was proven to be specific and sensitive for DNA amplification, with a sensitivity and specificity of 96.6% and 100%, respectively. This assay has potential for the rapid diagnosis of SPPV and GTPV in field diagnostic laboratories (Venkatesan et al. 2016; Mwanandota et al. 2018; Chala 2022). Zeedan et al. 2019  compared a variety of diagnostic techniques between serology and molecular methods to detect LSDV and its antibodies. The detection rate of qPCR was 39.13% greater than that of gel-based PCR. However, the FAT was the lowest at 32.6%. ELISAs detected 3.45% more LSDV antibodies than did IFATs.

D. Quantitative PCR (qPCR)

Rapid and accurate diagnosis of highly infectious viral diseases is key to controlling disease outbreaks in susceptible animals. Routine surveillance in accordance to rapid and sensitive molecular detection of the viral DNA/RNA is an important first step toward successful prevention and control (Shafagati et al. 2016; Xu et al. 2022). Nanotrap® Microbiome A Particles (NMAPs) are highly porous, thermostable hydrogel particles coupled with chemical affinity baits that able to concentrate a broad range of virions. It was able to capture, concentrate, and recover several highly infectious animal disease viruses, including GTPV, SPPV, LSDV and others. It was analyzed by virus-specific qPCR/RT-qPCR (Das et al. 2024).

While LSD is circulating in Egypt, regardless of climatic factors, using qPCR for accurate and rapid detection of the virus. In a recent study (Zeedan et al. 2024) authors used qPCR targeting P32, VP32, G protein, and viral fusion protein genes and compared commercial qPCR kit (LSDV dtec-qPCR kits; GPS™, Alicante, Spain) to rt-qPCR-SYBR Green. They concluded that rt-qPCR and multiplex PCR are needed for rapid routine diagnostic assays for LSDV from blood, tissue, and ticks’ samples. These results support the evolution of successful control measures.

#### Serological tests

Sera collected from acute and recovering animals were used for measuring antibodies against LSDV. Neutralizing antibodies appear approximately four days after the appearance of clinical symptoms and reach their final level in the 2nd or 3rd week. Complement-fixed and precipitated antibodies were detected in the serum of infected and convalescent animals (Lefèvre et al. 2010; WOAH 2023).

##### Virus neutralization test (VNT)

VNT is commonly used as a serological test to detect LSDV antibodies (Gari et al. 2008; WOAH 2023). The VNT measures the humoral immune response against LSDV in the sera of vaccinated calves using reference antisera, which confirms the presence of LSDV (Aboelkhair et al. 2019; WOAH 2023). The neutralizing titer was calculated according to (Reed and Muench 1938) and (Milovanović et al. 2020). They found that the commercially available ELISA kit from IDVet for the detection of capripoxvirus specific antibodies can detect antibodies as much lower as r ≥ 1.10 (Milovanović et al. 2020). Previously, VNT was considered valuable for studying antibody responses to infection (Davies 1982; House et al. 1990; Haegeman et al. 2020). VNT can be depended on to rule out false positives with pronounced specificity because of cross-reaction with cowpox and Parapoxvirus antibodies, but it is a time consuming difficult test to use for routine diagnostics (Davies and Otema 1981; Samojlović et al. 2019).

Among the serological tests for LSD diagnosis, the VNT remains the gold standard, as does ELISA (Datten et al. 2023). Cross-reactions between antibodies produced by other poxviruses play a role in lowering the specificity of AGID and IFAT assays over VNT. A capripox double-antigen ELISA was performed in the field and proved its superiority over SNT in terms of specificity; therefore, it was considered an ideal means to serologically differentiate between LSDV-vaccinated and infected cattle (Möller et al. 2019; WOAH 2023). According to (Maher 2015), the identification of LSDV antibodies in the two tests had the same specificity, with sensitivities of 96.2% and 98.7% for SNT and ELISA, respectively. However, compared with IFTA or VNT, ELISA has been proven by experimentation to have superior specificity and sensitivity (Zeedan et al. 2019; Milovanović et al. 2020).

##### Agar gel immunodiffusion test (AGID)

The AGID has been used for distinguishing the triggering antigen of CaPV but has the drawback of false-positive results due to cross-reaction with bovine papular stomatitis virus and pseudocowpox virus (Kitching et al. 1986; WOAH 2023).

##### Indirect immunofluorescence antibody test (IFAT)

The IFAT is a histochemical laboratory staining technique that utilizes the specificity of antibodies to their antigen. It is a broadly used method in immunohistochemistry because of the use of fluorochromes (WOAH 2023) to visualize antibodies. When the CaPV antigen is added to a cell culture plate, IFAT can detect antibodies against LSD in the serum. The test was found to have great sensitivity, but cross-reactive Parapox and Orthopox viruses might impact its specificity at lower serum dilution rates (WOAH 2023). IFAT was able to display specific intracytoplasmic yellowish-green fluorescent granules that are indicative of LSDV (El-Nahas et al. 2011; Ateya et al. 2017).

##### Enzyme-linked immunosorbent assay (ELISA)

Researchers were able to clone a highly antigenic CaPV structural protein, P32, where this recombinant antigen was used in the construction of diagnostic kits containing the P32 monospecific polyclonal antiserum and the production of monoclonal antibodies (MAbs) (Heine et al. 1999). Using hyperimmune rabbit antiserum produced by the inoculation of rabbits with purified CaPV, capripox antigen from biopsy samples or tissue culture supernatant can be blocked on an ELISA plate. Antibodies against LSDV were detected by ELISA in all examined samples from clinically infected cows, indicating overall seropositivity (Sthitmatee et al. 2023). A unique experiment was performed by (Milovanović et al. 2020) using ELISA to detect LSDV-specific antibodies in milk. This lead technology involves unintrusive sampling, in which a variety of samples can be assembled and used for substantial identification (Krešić et al. 2020).

Two recombinant truncated proteins were tested and expressed, the capripoxvirus homologs of the vaccinia virus C-type lectin-like protein A34 and the EEV glycoprotein A36, as antigens for an indirect ELISA (iELISA) to detect anti-capripoxvirus antibodies (Berguido et al. 2022). They optimized iELISA using two different working conditions on both LSD in cattle and SPP/GTP in sheep and goats. The assays were proved sensitive and specific in sheep, goat, and cattle sera.

Tian et al. (2010) selected two amino-acid sequence (residues 92–118 and 156–175) and used this sequence for synthesis of one 27 amino-acid and one 20 amino-acid synthetic antigen. These synthetic peptides were used in developing affordable ELISA, that offers high-throughput sero-surveillance on a herd basis.

Ebrahimi-Jam et al. (2021) aimed to develop an ELISA protocol based on the recombinant full-length and truncated P32 protein (Tr.P32) of goat pox virus which was expressed in Rosetta strain of E. coli using pET24a + vector and evaluated by SDS-PAGE and Western blotting. Tr.P32 was purified by Ni–NTA affinity chromatography under denaturing conditions and used to develop a capripoxvirus-specific ELISA. The diagnostic potential of the developed ELISA was evaluated using control sera collected from goat, sheep, and cattle. The recombinant Tr.P32 showed good reactions with antibodies against GTPV, SPPV, and LSDV, with recognized specificity, assured by neutralization index (NI), there was no cross-reactions with anti-Orf virus antibodies. These indicate validity of using such recombinant proteins in sero-surveillance of all capripoxviruses.

##### Western blot analysis (WBA)

WBA offers a sensitive and specific procedure for antibody detection against CaPV structural proteins, yet it is costly and challenging to perform (WOAH 2023).

#### Novel approach for diagnosing LSDV

##### Immunoperoxidase monolayer assay (IPMA)

**IPMA** is a novel assay created to detect LSDV antibodies in simple and low-tech laboratories. The assay was demonstrated to be highly sensitive, specific, and replicable. In parallel to standard serological assays, LSDV-IPMA was capable of detecting LSDV antibodies in vaccinated/infected animals as early as the start of the disease. Due to its high level of safety and ease of use, IPMA can be utilized in regular biosafety laboratories (Haegeman et al. 2020). This new assay has potential for LSD diagnosis. It is a cheap and convenient test and has higher sensitivity and specificity than VNT and ELISA (Bedeković et al. 2017; Haegeman et al. 2020)**.**

##### High-resolution melting (HRM)

HRM is a new technique based on real-time high-resolution fusing PCR (Pestova et al. 2018). It produces four distinct melting peaks, enabling segregation between SPPV vaccines, SPPV field isolates, GTPV, and LSDV and between LSD, bovine papular stomatitis, pseudocowpox and cowpox viruses (Gelaye et al. 2017). In addition, it was able to differentiate poxviruses belonging to the *Orthopoxvirus, Capripoxvirus*, and *Parapoxvirus* genera (Omoniwa et al. 2023). The DNA of the two viruses named LSDV and pseudocowpox virus (PCPV) were collected from the disease materials in situ was developed by PCR, and then, depending on the melting temperature of the generated amplicons, they were able to distinguish between the two viruses. The LSDV-ORF010 gene specifically targets LSDV and is characterized by its unique species specificity (Modise et al. 2021). Using HRM PCR amplification, found that the type of virus can be analyzed after genus-specific primers amplify sample viral DNA and bind dyes. This assay is sensitive, specific, and inexpensive for detecting and classifying CaPVs. It was able to distinguish in the field between three strains of sheep and goat pox virus (SPPV & GTPV) as well as vaccine strains of lumpy skin disease virus (LSDV) (Pestova et al. 2018; Chibssa et al. 2019). This advanced technique was used to produce an innovative quantitative real-time PCR for rapid differentiation between the vaccine and field strain(s) of LSDV (Kumar et al. 2023b). In a study (Erster et al. 2019), they succeeded to utilize and validate HRM analysis in line with RFLP to be valid differential assays that are suitable for routine diagnosis for all LSDV isolates examined either from infected or vaccinated animals. HRM primers were able to distinguish the fully conserved regions in all isolates in this study.

##### Recombinase polymerase amplification (RPA) assay

RPA is a novel rapid diagnostic technique for the LSDV-ORF068 gene utilized in combination with a CRISPR-Cas12a-based fluorescence assay (RPA-Cas12a-fluorescence assay). In an experiment conducted in Dakahlia Governorate, Egypt, in 2012 (Shalaby et al. 2016), the authors detected LSDV in trace amounts in the samples. The assay showed rapid, superior accuracy and sensitivity and could be used on farms or at quarantine points**.** It did not show mutual reactions with other widespread bovine viruses (Jiang et al. 2022).

##### Lateral flow immunoassay (LFIA)

LFIAs depend mainly on colorimetric sandwich-type LFIAs with rapidity and are recognized using two monoclonal antibodies against different epitopes of the P32 structural protein of LSDV and gold nanoparticles (Cavalera et al. 2022). Although its sensitivity is similar to that of ELISA, it has not been extensively used in field diagnosis, and its specificity needs to be verified through many clinical trials.

##### Easy express extraction (Triple***E***)

Korthase and colleagues (Korthase et al. 2022) approaches a fast and affordable method (*E*asy-*E*xpress-*E*xtraction, called Triple*E*) as a centrifugation-free and electricity-free nucleic acid isolation method. The procedure is based on the well-established magnetic-bead extraction technology using an in-house self-made magnetic channel and a rod cover. They extracted nucleic acid from 8 samples within 10 min without any instruments and confirmed the assay sensitivity. The novel method, point-of-care, was subsequently used extensively. This is in contrast with other abovementioned assays, which depend mainly on the availability of power equipment. The test can be applied in situ in the absence of good laboratory practice for diagnosis.

### Differential diagnosis

Clinical manifestations of LSD can be like other illnesses, leading to field doubts. Proficient preventative and control procedures in susceptible herds are guaranteed, and it is valuable to achieve an obvious diagnosis. Pseudo-LSD, which is caused by bovine herpesvirus type 2 (BoHV2), can cause nodule-like and skin swelling, similar to LSD. The virus usually has a milder clinical picture, revealing superficial nodules without systemic symptoms, as in the early stages of LSD. One straightforward difference is histopathological examination, where intranuclear inclusion bodies and viral syncytia can be found with bovine herpesvirus type 2 (BoHV-2) infection but not with LSD infection (Möller et al. 2019). Other differential diagnoses should be listed according to the lesions and their location on the animal. In terms of integumentary lesions, the following symptoms are observed: photosensitization, dermatophilosis, dermatophytosis, bovine farcy, actinobacilosis, actinomycosis, urticaria, insect bites, nocardiasis, besnoitiosis, demodicosis, onchocerciasis, cowpox, and pseudocowpox. Mucosal lesions can also include bluetongue, foot and mouth disease, malignant catarrhal fever, bovine viral diarrhea, bovine common stomatitis, and infectious bovine rhinotracheitis (Farah Gumbe 2018). On the other hand, several basic diagnostic methods can aid in differential diagnosis, one of which is inoculation of a susceptible sample in an embryonated chicken egg (ECE), which causes characteristic pitting lesions on chorioallantoic membranes (CAMs) (El-Ansary et al. 2022). Pathology of skin biopsies can be performed by immunohistochemistry (IHC) using specific anti-LSDV antibodies, and the distribution of viral antigens can be detected. Not only is antigen detection beneficial for pathological examination, but tissue changes in the skin layers after LSDV infection can also be observed microscopically. These changes include watery degeneration, granulomatous reactions, dystrophic calcification of the dermis, and the formation of inflammatory cells (Sanz-Bernardo et al. 2020; Amin et al. 2021). Histopathological examination revealed inclusion bodies in the cytoplasm of bovine skin capsules and confirmed that these inclusion bodies were a typical pathological feature linked to LSD (Ali et al. 2021).

### Distinguishing between wild-type and vaccine viruses of LSDV and recombinant LSDV viruses

Using three methods, (Menasherow et al. 2014) were able to differentiate between vaccine and wild-type strains. First, genetic sequencing of the enveloped virion (EEV) gene was shorter by 27 bases in the Neethling vaccine strain than in the Israeli virulent strain. The second method utilizes a traditional PCR assay involving the use of specific primers, amplification of the virus genome by upstream and downstream primers and nested PCR. The authors were able to ascertain the molecular fingerprint of the virus through differences in the annealing temperatures. The third step involves digesting the PCR product through the Mbo I enzyme, which can digest the vaccine strain but not the virulent strain. Clinically, the other two methods are more rapid and widely used (Menasherow et al. 2014; Agianniotaki et al. 2017) and exploit a dual real-time PCR method for identifying GPCR genes. The amplification efficiencies of the wild and vaccine viruses were 91.3 and 90.7%, respectively. To date, several identification assays that have been developed need to analyze the data to obtain the results. Möller et al. (2019) used real-time qPCR to distinguish between virulent and vaccine strains with 100% accuracy. On the other hand, by utilizing the EEV gene, (Agianniotaki et al. 2021) developed a unique method, duplex real-time PCR, which was able to specifically reveal the presence of wild-type LSDV in samples loaded with a high-titer LSDV vaccine. The amplification efficiencies of the viruses were 99.0% and 98.6% in the presence and absence of the LSDV, respectively. β-Actin was used as an internal amplification control to normalize the expression of the target gene between different samples (Table 2).

**Table 2:** Methods for distinguishing between vaccine and wild-type viruses of LSDV (Liang et al. 2022)

Method	Targets molecule	Accuracy	References
Nucleic acid sequence detection	Enveloped virions (EEV) gene	Unknown	(Menasherow et al. 2014)
Duplex real-time PCR		Unknown	(Agianniotaki et al. 2021)
Nested PCR	Genome of virus	Unknown	(Menasherow et al. 2014)
PCR	MboI enzyme cleavage site	Unknown	(Menasherow et al. 2014)
Dual real-time PCR	GPCR genes	Unknown	(Agianniotaki et al. 2017)
Double-stranded real-time qPCR	TaqMan probe	100%	(Möller et al. 2019)

Identification of LSDV as a vaccine or a field virus in vaccinated cattle triggered developing several molecular assays. The currently available real-time PCR assays to differentiate between infected and vaccinated animals (DIVA) have not been assessed for use with recombinant viruses. Byadovskaya et al. (2021) compared the diagnostic performance of two commercial real-time PCR DIVA kits and published DIVA assays against a panel of field, vaccine and vaccine-derived recombinant field isolates of LSDV. Previous isolates were used including vaccine-like isolates and recombinant Neethling vaccine-derived isolates. The study indicated that recombinant LSDVs cause improper LSDVs identification as either vaccine or field viruses. This illustrates the importance of accuracy in DIVA testing and that the currently available DIVA assays are not accurate when recombinant LSDV isolates are present.

## Control and prevention of LSDV

### Treatment of LSDV

An effective treatment for LSD has not been identified. The most common treatment protocol includes the use of broad-spectrum antimicrobial agents, along with anti-inflammatory and antihistamine agents, to mitigate secondary bacterial infections (Datten et al. 2023). The medications listed in (Table 3) were applied as symptomatic treatments, and the major goal was to save the animals' lives and avoid LSD. Antibiotics should be used for 5–7 days depending on the severity of the condition, along with careful care and treatment, to limit secondary infection (Zewdie 2021). Inoculation with vitamins such as B-complex and AD_3_E is considered a valuable supportive therapy that can maintain feeding capacity and reproductive preservation (Zewdie 2021). Recently, clinically infected animals were treated with alginate (Alg) propolis nanoparticles (NPs), which has proven to be an effective specialty medicine for the treatment of viral symptoms (Farag et al. 2020). Methylene blue (MB) is a broad-spectrum antiviral medication with well-known antiviral activity against a wide range of viruses. Through its multi-mechanism antiviral effect, MB will aid in the treatment of LSD (Shrirame et al. 2023).

**Table 3:** Therapeutic Agents for LSD Treatment

Therapeutic Agents	Pharmacological Effects	References
Enrofloxacin	Antibiotic	(Anil and Durga 2021; Islam et al. 2021)
Oxytetracycline		(Feyisa 2018; Anil and Durga 2021)
Penicillin		(Feyisa 2018)
Cephalosporin		
Tetracycline		
Fluoroquinolone		
Chlorpheniramine Maleate	Antihistamines	(Anil and Durga 2021; Islam et al. 2021)
Meloxicam	Nonsteroidal anti–inflammatory	
Dexamethasone Suspension	Steroidal anti–inflammatory	(Feyisa 2018)

### Measures for the prevention of lumpy skin disease

Quarantine, cleaning, and disinfection of contaminated locations, vector control and diagnosis of asymptomatic animals, carcass disposal and discarding of infected animals have all been used to eradicate LSDV outbreaks, but initiating vaccination programs prior to viral entry is a crucial part of eradication strategies for some significant outbreaks (EFSA 2018; WOAH 2023).

#### Restrictive movement

The movement of infected animals with LSD from one zone to another must be restricted to avoid the spread of the disease. Within countries, animals should be quarantined for extensive inspections if they show such lesions to prevent the rapid spread of the disease. Fundamentally, animal handlers and workers dealing with affected animals should be advised to keep away from healthy animals. Additionally, people’s movement should be restricted to different areas.

#### Restricting vector movements

As vector movement plays a great role in the spread of LSD due to winds, such vectors must be under restricted control methods; for instance, the use of specific mosquito traps and specialized insecticides can greatly aid in preventing the disease.

#### Networking and information

There are a great impact of mass communications and social networking in increasing the understanding and learning of the epidemiological studies, fast diagnosis, and speedy announcement of the disease spread.

#### Semen control measures

It is important to enhance awareness about LSD and encourage the use of vaccines for its prevention and control. Additionally, adopting effective biosecurity practices and improving herd management could help decrease the incidence of LSD in the region, thereby reducing the economic impact on farmers (Carn and Kitching 1995; Hailu et al. 2014).

## Research advances in LSD vaccines

Currently, vaccination appears to be the most effective strategy for managing LSD. In addition, there is a need to produce effective and safe vaccines**.**, The vaccine approach includes live attenuated vaccines, inactivated vaccines and live attenuated vectored vaccines (Francis 2018; Wang et al. 2020).

The Indian Veterinary Research Institute (IVRI) in Mukteswar has made significant strides in the development of a vaccine for Lumpy Skin Disease (LSD), which is caused by the Lumpy Skin Disease virus (LSDV). The vaccine, a live attenuated strain, has shown promising results in inducing protective immunity in cattle. Field trials have demonstrated its effectiveness in reducing the incidence and severity of LSD outbreaks, making it a vital tool in controlling this economically impactful disease in livestock. Alongside vaccination efforts, current diagnostic tests for LSD have evolved, with techniques such as polymerase chain reaction (PCR) and enzyme-linked immunosorbent assay (ELISA) playing crucial roles. These tests enable rapid and accurate detection of LSDV, facilitating timely intervention and control measures to curb the spread of the disease (Kumar et al. 2023a, b).

### Live-attenuated vaccines

Live attenuated vaccines refer to genuine active viral strains whose virulence has decreased through various laboratory procedures and treatments (Abdelrahman et al. 2020; Liang et al. 2022). Prevention by vaccination with attenuated virus is the most promising method for controlling the spread of the disease (Das et al. 2021; Bazid et al. 2023). However, live attenuated vaccines cannot be used in disease free regions without losing disease free status (Lee et al. 2012; Sprygin et al. 2018; Krotova et al. 2022). According to OIE recommendations, different strains of LSD virus were utilized commercially to produce different vaccines. It is established in Neethling strains such as the LSD Vaccine for Cattle (Onderstepoort Biological Products; OBP, South Africa), Bovivax (MCI Sante Animale, Morocco), and the SIS Neethling type (Lumpyvax, MSD Animal Health-Intervet, South Africa). Furthermore, vaccines against Sheeppox (SPP) and Goatpox (GTP) have to be used for LSD (Tuppurainen et al. 2015), as they share more than 97% similarity with their viruses (Tulman et al. 2001). The Neethling strain-based vaccine, which is the most common strain of LSDV, was isolated in South Africa occurred in Botswana in 1943 and South Africa in 1945; subsequently, it was purified and called the Neethling type (Hunter and Wallace 2001). These vaccines are currently being manufactured in South Africa.

The Neethling vaccine was four times more effective than the SPP vaccine for preventing laboratory-confirmed disease, with a relative vaccine efficacy of 77%. Full protection is provided by this vaccine approximately one month after vaccination (Tekilegiorgis 2019). However, according to the (EFSA 2017), > 1% of the livestock in Croatia produced adverse reactions when vaccinated with the Neethling vaccine (EFSA 2017, 2018; Calistri et al. 2019). In addition, the Ethiopian Neethling vaccine failed to protect against the disease (Gari et al. 2015) likely due to production issues leading to poor quality of the vaccine. (Bedeković et al. 2017) detected the vaccine virus in milk collected from vaccinated cows. Hence, vaccines should be fully assessed to obtain a targeted immune profile. (Haegeman et al. 2021) conducted several clinical trials comparing LSDV homologous live attenuated vaccines, including LSDV (South Africa), Lumpyvax (South Africa), Kenyavac (South Africa), Herbivac LS (South Africa) and Vaccine LSD Neethling O Vivant (Morocco). They recorded different counteractions, such as fever, but none of the former vaccines altered feed intake or daily health performance. Another field study showed that Neethling vaccine is significantly effective in preventing LSD morbidity (Ben-Gera et al. 2015). **The homologous live attenuated virus vaccine—the Neethling** strain—passed the virus 60 times in lamb kidney cells and 20 times on chorioallantoic membranes (CAMs) of embryonated chicken eggs (ECEs), potentially leading to immunity for up to three years especially when a booster vaccination was given 12 months after the initial vaccine (Tuppurainen et al. 2021). **The heterologous live attenuated virus vaccine** – sheep or goat pox vaccine – includes KSVP passaged 18 times in lamb testis (LT) cells or fetal calf muscle cells, the Yugoslavian RM 65 SPP strain, and the Romanian Sheep Poxvirus (RSPV) strain but may cause local, sometimes severe, reactions. However, it is not advised to establish vaccines for sheep and goats in countries free of Sheeppox (SPP) and Goatpox (GTP), as such vaccines may serve as a source of infection for susceptible herds of sheep and goats (Tuppurainen et al. 2021).

From 2006 to 2007, the Romanian strain 65 (RM65)-SPP vaccine was used to vaccinate cattle against LSDV at the same dose as advised for sheep, resulting in incomplete protection (Brenner et al. 2009). In the field, the lowest dosage of the SPP RM65 vaccine that provides protection against LSD should be used, as no systemic reactions are expected (Abutarbush and Tuppurainen 2018). In Turkey, the Bakirkoy SPPV was used in cattle against the LSD virus at three to four times the authorized dose for sheep, and there was no significant difference in disease incidence between cattle immunized with this vaccine and nonvaccinated cattle (Şevik 2017).

In Egypt, since the 1990s, Egyptian medical professions have experienced outbreaks of LSD caused by the use of a vaccine against a Romanian poxvirus strain (Ali et al. 1990). (Gari et al. 2015) verified that a KSGP 0–180 strain vaccine prepared in Kenya did not offer LSDV protection in cattle. **The Kenyan sheep and goat pox (KSGP) virus vaccines O-240 and O-180** have been used effectively as vaccines for SPP and GTP (Davies and Mbugwa 1985; Kitching et al. 1987). (Bamouh et al. 2021) confirmed that the KSGP O-180 and KSGP O-240 vaccine strains may cause vaccine virus shedding, resulting in infection of other unvaccinated or healthy cattle. According to field findings from Kenya, KGSP-180 vaccination is both safe and effective (Davies and Mbugwa 1985). During the 2006 LSD epidemic in Egypt, it was found that the live attenuated KSGP O-240 strain could not confer full LSD protection in cattle (Salib and Osman 2011). In Ethiopia, LSD outbreaks are allowed to occur due to the inferior performance of the native KSGP vaccine and insufficient vaccination coverage (Gelaye et al. 2017). In Oman, vaccination of cattle with the KSGP vaccine O-240 was not successful (Mathan Kum 2011). The KSGP virus vaccines O-240 and O-180 have been used successfully as vaccines for SPP and GTP (Davies and Mbugwa 1985; Kitching et al. 1987). **The Georgian goat pox virus (GGPV)** obtained by the Jordan Biological Center was used to counteract GTPV in the Middle East in 2010, (Abbas et al. 2010) and then (Gari et al. 2015) used it to treat LSDV, resulting in its significant stimulation of cellular immunity in vaccinated cattle, proving its immunogenicity against LSDV. **The GGPV vaccine** is considered a suitable vaccine for disease control in the field because it elicits a high antibody titer and high IL-4 production, as well as increased lymphocyte proliferation and IFN production, against the LSD virus (Varshovi et al. 2017). Contagious bovine pleuropneumonia (CBPP) is caused by *Mycoplasma mycoides subsp. mycoides* (Mmm), where (Safini et al. 2022) formulated a bivalent vaccine through attenuation of CBPP (a strain obtained from CIRAD AF262936) and the LSD-Neethling strain (ID: AF409138). These patients ultimately exhibited the production of measurable neutralizing antibodies against the two pathogens without any clinical adverse signs. It was concluded that this bivalent vaccine was able to protect against these two diseases. The occurrence of homologous recombination of double-stranded DNA viruses is extreme, and the original effect of the vaccine may not be achieved due to the increased virulence of the virus after inoculation and other viruses of the *Poxviridae* family. Thus, in the field of live attenuated vaccines, specific obstacles should be investigated in detail, and more appropriate vaccines should be selected according to the actual circumstances of the cows being vaccinated (Table 4). In Egypt, both the Romanian sheep pox virus (RSPV) and Kenyan sheep and goat pox (KSGP) virus vaccines have been used for cattle (Brenner et al. 2009; Abutarbush 2014; Tuppurainen et al. 2017).

**Table 4:** Live Attenuated Vaccines for LSD (Liang et al. 2022)

Strain	Virulence	Virus Titer	Adverse reaction after vaccination	Challenge	Protection	Reference
South Africa “Neethling”	Low	104.5 TCID50	50% of vaccinated cattle had swelling at the inoculation site	Do not verify	Do not verify	(Weiss 1968)
Ethiopian “Neethling”	Low	103.5 TCID50	No adverse reactions	Ethiopia LSDV-wild type	30%	(Gari et al. 2015)
South Africa “Neethling” (Pirbright Institute)	Low	104.0, 105.0 TCID50	6.7% of vaccinated cattle showed Neethling disease	Do not verify	Do not verify	(Bamouh et al. 2021)
KSGP O-180 (Kenyan sheep and goat pox)	Low	104.5, 103.5 TCID50	No adverse reactions	Ethiopia LSDV-wild type	50%	(Gari et al. 2015)
KSGP O-240 (Kenyan (Kn) Sheep and Goat Pox by Pirbright Institute)	Low	104.0, 105.0 TCID50	3.7% of cattle vaccinated with low doses showed Neethling disease; 11.9% of cattle vaccinated with high doses developed skin lesions	Do not verify	Do not verify	(Bamouh et al. 2021)
Gorgan GTP (Jordan Bio-Industries Centre (JOVAC))	Low	104.5, 103.5 TCID50	No adverse reactions	Ethiopia LSDV-wild type	100%	(Gari et al. 2015)
South Africa Neethling (Onderstepoort Biological Products SOC Ltd.)	Low	103.5 TCID50	12% of cattle developed lumps at the inoculation site; 9% of animals developed small lumps at the inoculation site after 8–18 days; Vaccine virus can be detected in milk from vaccinated cows	Do not verify	Do not verify	(Bedeković et al. 2017; Katsoulos et al. 2017)
Homologous strain	Unknown	Unknown	0.09% of vaccinated animals developed fever, injection site edema, and decreased milk production	Do not verify	Do not verify	(EFSA 2018)
Lumpyvax (MSD Intervet South Africa (Pty) Ltd., Spartan, RSA, attenuated SIS type virus)	Low	104.0 TCID50	Vaccine virus can be detected in milk from vaccinated cows	Do not verify	Do not verify	(Bedeković et al. 2017)
Onderstepoort (Biological Products OBP; South Africa; batch 442)	Low	Unknown	86% of cattle showed hypothermia after vaccination	LSD/OA3-Ts.MORAN	100%	(Haegeman et al. 2021)
Lumpyvax (MSD-Animal Health; South-Africa; batch BNDM07)	Low	Unknown	All cattle exhibited hypothermia after vaccination	LSD/OA3-Ts.MORAN	100%	(Haegeman et al. 2021)
Kenyavac (Jordan Bioindustries Center Jovac; Jordan; batch 220,115–04)	Low	Unknown	71% of vaccinated cattle showed hypothermia after vaccination	LSD/OA3-Ts.MORAN	100%	(Haegeman et al. 2021)
Herbivac LS (Deltamune; South-Africa)	Low	Unknown	Vaccinated cows had enlarged prethoracic lymph nodes; 57% of vaccinated cattle showed hypothermia after vaccination	LSD/OA3-Ts.MORAN	100%	(Haegeman et al. 2021)
Vaccine LSD Neethling O vivant (MCI Santé Animal; Morocco, batch 17BLSDN001)	Low	Unknown	43% of vaccinated cattle had severe swelling greater than 10 cm in diameter at the inoculation site; 57% of vaccinated cattle showed hypothermia after vaccination	LSD/OA3-Ts.MORAN	100%	(Haegeman et al. 2021)
RM65 (Abic Ltd. Netania, Isral)	Low	103.9 TCID50	11.1% of the vaccinated cattle developed typical symptoms of LSD	Do not verify	Do not verify	(Brenner et al. 2009)
combined Mmm/LSDV vaccine	Low	104.5 TCID50 for LSDV 108 CCU50 for Mmm	No adverse reactions	Do not verify	Do not verify	(Safini et al. 2022)

### Live attenuated vectored vaccines

Recombinant vectored vaccines are live attenuated virus vaccines modified to express genes encoding antigens to elicit protective immunity. Many viral vectors have a limited capacity to express foreign antigens. Therefore, it is important to select the best antigen(s) to elicit protective immunity following vaccination (Teffera and Babiuk 2019).

Vaccination for LSD is accomplished in a bi or multivalent-vectored vaccine based on LSDV has advantages compared to conventional vaccination. LSD-vectored candidate vaccine expressing the critical glycoproteins of Rift Valley fever virus (RVFV) was developed in a collaboration project between the Agricultural Research Council-Onderstepoort Veterinary Institute (ARC-OVI) and Onderstepoort Biological Products (OBP Ltd). This bivalent LSD-RVF vaccine has been shown full protection in both lab and field animals against virulent RVFV challenge (Wallace et al. 2006).

The advantages of using a bivalent marker free LSD-RVF.mf vaccine over conventional vaccination include: (1) a vaccine which is cost effective; (2) since RVF outbreaks are cyclical, producers are reluctant to vaccinate as it is expensive, and thus having a bivalent capripoxvirus vaccine removes these economic reasons for not vaccinating; and (3) the bivalent vaccine is able to differentiate infected from vaccinated animals (DIVA) for RVF, allowing for serological surveillance testing to still be performed when it is in use. The impact of using the bivalent LSD-RVF.mf vaccine includes: (1) preventing mortality and debilitating disease in cattle caused by LSD and RVF in all regions of sub-Saharan Africa where the diseases both occur, leading to improved production and economic development; (2) indirectly protecting people from RVF virus by decreasing the viral loads in livestock; and (3) helping provide an effective barrier to further spread of RVFV into non-endemic countries (Wallace et al. 2020).

### Inactivated vaccines

Inactivated vaccines involve the inactivation of complete viruses through physical, chemical, and biological methods. This process ensures that viruses are effectively killed and lose their infectivity and virulence while preserving their ability to stimulate an immune response. Inactivated vaccines have several advantages, including low production cost, a short development cycle, and good efficacy. Unlike live attenuated vaccines, inactivated vaccines usually require booster immunization to provide protection against virus invasion (Bhanuprakash et al. 2004). Various antibodies can be detected, and studies have shown that the antibody response rate to inactivated vaccines by VNT is 37% greater than that to live attenuated vaccines on the 28th day postvaccination (Hamdi et al. 2020). In 2020, (Es-sadeqy et al. 2021) utilized a bivalent inactivated vaccine with oil adjuvants against LSDV and Bluetongue virus (BTV). This vaccine initiated the production of high levels of neutralizing antibodies and improved animal welfare and ethics by excluding the need for challenge tests. However, further experiments are needed to validate its specific clinical effectiveness. (Wolff et al. 2020) emphasized that different vaccine adjuvants can enhance the effectiveness of inactivated vaccines. They used two adjuvants, adjuvant A and adjuvant B, in the inactivated Neethling vaccine. Adjuvant A, which contains a low molecular weight copolymer, effectively stimulated the humoral immune response and the production of IFN-γ in vaccinated cattle, making it an ideal adjuvant in clinical settings. (Matsiela et al. 2022) used a low concentration of BEI to inactivate the Neethling strain and employed Montanide™ Gel-01 as a vaccine adjuvant. They successfully generated a high level of neutralizing antibody in immunized rabbits. Although this approach has not been tested in cattle, the newly developed adjuvant can serve as a reference for future studies. One limitation of inactivated vaccines is that they tend to elicit a narrow immune response. While they are effective at persuading antibodies, they are less capable of inducing cell-mediated and mucosal immune responses. Therefore, there is a need to develop a safer and more efficient inactivated vaccine against LSDV (Table 5).

**Table 5:** The Inactived Vaccines of LSD (Liang et al. 2022)

Strain	Virus Titer	Adjuvant	Adverse Reaction	Challenge	Protection	Reference
South Africa “Neethling”	106.0 TCID50	Oil	No adverse reactions	Virulent LSDV Israeli field isolate	100%	(Hamdi et al. 2020)
LSDV-BTV4	106.0 TCID50	Oil	No adverse reactions	BTV4	LSDV do not verify; BTV 100%	(Es-sadeqy et al. 2021)
LSDV- “Neethling Vaccine”	107.0CCID50	Low molecular	No adverse reactions	LSDV “Macedonia2016” field strain	100%	(Wolff et al. 2020)
LSDV- “Serbia” field strain	106.0CCID50; 107.0CCID50	A combination of Amphigen, Quil A and Cholesterol	Presents as mild Neethling disease	LSDV “Macedonia2016” field strain	100%	(Wolff et al. 2020)
OBP- “Neethling Strain	106.0TCID50	Montanide™ Gel-01	-	-	-	(Matsiela et al. 2022)

### Recombinant modified live virus vaccines

Live attenuated vaccines are considered the best method because they retain all relevant antigens and allow the pathogen to replicate within the host. This replication process stimulates both cellular and humoral immunity in the host. However, there is a risk of the attenuated virus regaining its virulence. To address this concern, scientists have attempted to identify the virulence genes of various pathogens. They have employed directed mutation or deletion of these virulence genes to deliberately attenuate the pathogen and create a recombinant attenuated strain (Liang et al. 1991). In sheep pox, the Kelch-like gene SPPV-019 has been demonstrated to attenuate a virulent SPPV (Balinsky et al. 2007). This recombinant **live attenuated** vaccine candidate thus has the potential to be used in ruminants as a cost-effective vaccine against LSD (Aspden et al. 2002). The South African LSDV vaccine strain shows good potential for use as a vector for recombinant vaccines using the viral thymidine kinase (TK) gene as a foreign gene insertion site (Wallace and Viljoen 2005). A method for generating recombinant CaPVs by homologous recombination and further testing a CaPV vector vaccine to deliver foreign antigens has been described (Liu et al. 2019). Heffner and Peeters (Heffner et al. 1993; Peeters et al. 1994) focus on ensuring safety has opened up new possibilities for vaccine preparation through the recombination of LSDV with other viruses. They identified the recorded recombinant vaccines worldwide (Table 6). Some vectors were modified by gene insertion, Kenya sheep-1 (KS-1) either by one or two genes. While others were modified by deletion, LSD OBP vaccine (ht-LSD-OBP). All showed a full protection rate which indicates their efficacy as well as safety.

**Table 6:** Recombinant Live Attenuated Vaccines for LSD (Liang et al. 2022)

Vector Strain	Insert Genes	Delete Genes	Challenge	Protection Rate	References
Kenya sheep-1(KS-1)	Fusion(F) protein-encoding gene of RPV	-	Virulent lumpy skin disease virus	100%	(Romero et al. 1993)
Kenya sheep-1(KS-1)	Hemaglutinin and fusion protein genes of RPV	-	Virulent Neethling strain	100%	(Ngichabe et al. 2002)
			Kabete O strain	55%	
LSD OBP vaccine (ht-LSD-OBP)	Open reading frame (ORF) 005	Interleukin-10-like (IL-10) gene	LSDV-WB	100%	(Kara et al. 2018)
LSD OBP vaccine (ht-LSD-OBP	ORF 008	Interferon gamma receptor-like (IFN-γR) gene	LSDV-WB	100%	

## Conclusion

Cattle have traditionally been considered the primary host for LSDV, leading to a significant decline in the meat industry. In clinical applications, different diagnostic procedures can be chosen based on the phase of the disease. In the early stages of LSDV infection, modern molecular techniques are highly sensitive, and antibody level assays can be used. Early detection and treatment are crucial for preventing and managing this disease effectively. As clinical symptoms become apparent, typical pathological abnormalities can aid in differential diagnosis. Both PCR and LAMP tests have shown sensitivity in identifying the causative agent of LSDV. Commercially available CaPV double-antigen ELISA has demonstrated comparable specificity to SNT, making it a useful method for quick and convenient serological detection of LSDV in vaccinated or infected cattle. Control measures against LSD include disinfection of contaminated facilities, immunization, vector control, identification of subclinically infected animals, proper carcass disposal, quarantines, and movement control. Additionally, there is a need to develop novel vaccines that are both effective and safe. CaPV-attenuated strains have been used as vaccines to combat LSD. Recombinant vaccine candidates hold promise for use in cattle as low-cost, safe, effective, and stable immunizations against LSD. Killed LSD vaccines may also be beneficial for live cattle export and import. To date, attenuated CaP vaccination has been utilized in Egypt. Extensive clinical studies are required to validate the effectiveness of established preventive and diagnostic approaches. Therefore, future efforts will focus on disrupting effective transmission pathways, implementing large-scale safe and efficient vaccination strategies, and employing appropriate diagnostic procedures.

## Recommendations

Apart from typical clinical signs, it is important to determine the hematological and biochemical clinical profiles of cattle affected by LSD. Accurate and timely diagnosis is crucial for implementing control measures. Long-term mandatory vaccination with 100% coverage with an effective LSDV vaccine is necessary to effectively control and prevent LSD, as the virus is stable and can persist in the environment for extended periods of time. Only homologous LSDV live attenuated vaccines have been effective in the field to control and eradicate LSDV. Animals must be vaccinated before being introduced to an affected farm. Immunization at 3 to 4 months of age, preferably from vaccinated or naturally infected mothers. Pregnant cows and breeding bulls can be vaccinated yearly. In financially constrained remote regions, complete vaccination and disease elimination may not be feasible, but at the very least, vaccinated cattle should be permanently indicated. Vaccinated and unvaccinated cattle can be kept together, utilizing herd immunity to lower spates. Even after vaccination, some animals may still experience illness. It is crucial to determine whether an animal is infected with a wild type virus or a vaccine strain. Since LSDV is transmitted by insects, studying the possibility of using antiparasitic medications in conjunction with vaccinations to effectively prevent LSDV transmission is important. During periods of active insect migration, vector management and restrictions on animal movement play a critical role.

## Data Availability

No datasets were generated or analysed during the current study.
